# Combined treatment of umbilical cord Wharton’s jelly-derived mesenchymal stem cells and platelet-rich plasma for a surgical patient with hospital-acquired pressure ulcer: a case report and literature review

**DOI:** 10.3389/fbioe.2024.1424941

**Published:** 2024-07-09

**Authors:** Changhui Zhou, Linlin Jiao, Xiaoping Qiao, Weiwei Zhang, Shuangfeng Chen, Chunling Yang, Min Meng

**Affiliations:** ^1^ Department of Central Laboratory, Liaocheng People’s Hospital, Liaocheng, China; ^2^ Nursing Department, Liaocheng People’s Hospital, Liaocheng, China; ^3^ Department of Traditional Chinese Medicine, Liaocheng People’s Hospital, Liaocheng, China

**Keywords:** hospital-acquired pressure ulcers, mesenchymal stem cells, platelet-rich plasma, treatment, transplantation

## Abstract

Hospital-acquired pressure ulcers (HAPUs) are still an important worldwide issue related to the safety and quality of patient care, which are among the top five adverse events reported. Patients who develop HAPUs have longer stays in the hospital than necessary, are at a greater risk of infections, and are more likely to die. Surgical patients are prone to developing PUs because they often remain immobile for extended periods of time, and their surgical procedures may limit the flow of blood oxygen and nutrition and lead to a decrease in muscle tone. Mesenchymal stem cells (MSCs) represent an attractive stem cell source for tissue regeneration in clinical applications, which have been demonstrated to improve wound healing through re-epithelialization, increased angiogenesis, and granulation tissue formation. Here, we present the case of an emergency surgical patient who developed an ulcer on the right heel during hospitalization. The human umbilical cord Wharton’s jelly-derived MSCs (WJ-MSCs) re-suspended in platelet-rich plasma (PRP) were injected into ulcer margins. Four days after the WJ-MSC application, the patient showed progressive healing of the PU. From days 4 to 33, granulation tissue formation and re-epithelialization were clearly observed. The ulcer was almost healed completely on day 47, and the pain in the patient’s wound area also decreased. Thus, intradermal transplantation of WJ-MSCs and PRP was safe and effective for treatment in patients with pressure ulcers. WJ-MSCs, together with PRP, may offer a promising treatment option for wound healing.

## Introduction

Pressure ulcers (PUs), also known as pressure injuries, pressure sores, or bedsores, are defined as a type of injury that breaks down the skin or underlying soft tissues, usually over a bony prominence or related to a medical or other device (Gillespie et al., 2014; Chaboyer et al., 2016). The injury usually occurs as a result of intense and/or prolonged pressure or pressure in combination with shear (Gaspar et al., 2019). With the aging of the population worldwide, the number of patients with PUs is increasing every year (Mervis and Phillips, 2019). Hospital-acquired PUs (HAPUs) in the hospital context remain an important worldwide issue related to the safety and quality of patient care, ranking among the top five adverse events reported (Brandeis et al., 2001; Gillespie et al., 2014). The incidence and prevalence rates of PUs among hospitalized patients vary greatly, ranging from less than 3% to over 30% (Nixon et al., 2006; Schuurman et al., 2009). Once PUs form, conservative management is usually indicated for less severe ulcers (stages I and II); the current therapeutic approaches for severe ulcers (stages III and IV) are surgery and negative pressure (Cushing and Phillips, 2013). Given the current challenges in prevention and management strategies for PU patients, new perspectives, technologies, and more effective treatments must be further considered.

With the development of stem cell therapy and tissue engineering in recent years, mesenchymal stem cells (MSCs) have shown promising outcomes in treating chronic wounds such as pressure ulcers, radiation-related skin injuries, severe skin burns, and non-healing diabetic ulcers (Portas et al., 2016; Pelizzo et al., 2018; Wu et al., 2018; Han et al., 2019; Torres-Guzman et al., 2023). The main sources are umbilical cord-derived MSCs (UC-MSCs), bone marrow-derived MSCs (BM-MSCs), and adipose tissue-derived MSCs (AD-MSCs) (Wang et al., 2016). Among these, the umbilical cords are easier to obtain and culture MSCs due to noninvasive collection, cost-effectiveness, productivity, and ethical access (Arno et al., 2014). In particular, the umbilical cord Wharton’s jelly-derived mesenchymal stem cells (WJ-MSCs) have been demonstrated to be young, immunomodulatory, and non-tumorigenic cells with lower immunogenicity, faster proliferation, and greater *ex vivo* expansion capabilities than other human MSC sources, such as BM-MSCs and AD-MSCs (Nekanti et al., 2010). Arno *et al.* proved that human WJ-MSCs can promote wound healing by paracrine signaling in an excisional full-thickness skin murine model (Arno et al., 2014). WJ-MSCs possess the capacity to be induced to differentiate into sweat gland-like cells, which would be potential in sweat gland restoration after skin injury and improvement in cutaneous regeneration (Xu et al., 2012). Meanwhile, WJ-MSCs decrease liver, kidney, and lung fibrosis, which play a more important role in promoting skin regeneration (Moodley et al., 2009; Tsai et al., 2009; Du et al., 2012). Thus, the clinical application of WJ-MSCs has been shown to accelerate wound healing, and they have great potential in treating PUs.

In recent years, platelet-rich plasma (PRP), also named autologous platelet gel, has raised a great deal of interest in promoting angiogenesis and increasing the healing rate of PUs (Volakakis et al., 2019; Uçar and Çelik, 2020). It is obtained from the peripheral blood of the patient with elevated concentrations of platelets and extensively adapted and applied to acute and chronic wounds (Xiao et al., 2021). Recent findings proposed that platelets, growth factors, cytokines, chemokines, and fibrin in PRP gel can aid in wound healing and skin regeneration by interacting with fibroblasts, promoting the production of collagen fiber, facilitating cell recruitment, and increasing keratinocyte migration (Martínez et al., 2015; Hu et al., 2024). The use of PRP, especially in combination with MSCs, may present therapeutic potential in the treatment of wound healing through preserving or enhancing the tissue regenerative properties of transplanted cells (Tang et al., 2015). Many studies have proved PRP has the optimal effect on the growth of BM-MSCs and AD-MSCs (Kocaoemer et al., 2007; Cervelli et al., 2012; Murphy et al., 2012). In addition, PRP could prolong the survival and retention of transplanted MSCs, which may be a powerful tool to attract cell populations in the wound area (Lamo-Espinosa et al., 2020). Therefore, MSCs, together with PRP, may be an advantageous approach to treating PUs.

Despite the increasing availability of WJ-MSCs or PRP for improving and accelerating wound healing, the therapeutic effects of the combination of WJ-MSCs and PRP on PUs are still rarely reported and need to be confirmed. With the aim of developing a more effective therapeutic regime for PUs in the future, we present a case of an emergency surgical patient with a PU on the right heel. The purpose of this study is to present a review of the existing literature on PUs, and the clinical outcome of combination therapy will be critically discussed to explore their therapeutic potential for the patient.

## Case presentation

The study was conducted at Liaocheng People’s Hospital and approved by the Ethics Committee of Liaocheng People’s Hospital (2021094). This report was conducted in accordance with the CARE (CAse REport) guidelines.

On 3 July 2023, a 49-year-old man was admitted to emergency surgery at Liaocheng People’s Hospital because of multiple trauma to the right lower limb. Three hours before his admission to our hospital, the patient was accidentally injured by a steel plate impact during the work process. Therefore, the patient was admitted to the hospital for an emergency surgery operation. Past medical history includes good health with no history of diabetes, hypertension, heart disease, or cerebrovascular disease. The patient denied a history of hepatitis, tuberculosis, and other infectious diseases, as well as any history of surgery, trauma, blood transfusion, or drug allergy. The physical examination showed a body temperature of 36.7°C, blood pressure of 105/60 mmHg, and a pulse of 107 beats per minute. The admission X-ray showed a comminuted fracture and soft tissue injury of the proximal right femur and right distal femoral fracture. Laboratory examinations: Table 1 shows the patient’s laboratory test results after admission to our hospital. The levels of C-reactive protein and interleukin-6 (IL-6) were significantly elevated.

**TABLE 1:** Laboratory test results of the patient at admission.

Characteristic	Result	Range
White blood cell (10^6^/L)	12.97	3.5–9.5
Red blood cell (10^12^/L)	2.76	4.3–5.8
Hemoglobin (g/L)	82	130–175
Platelet (10^9^/L)	109	125–350
Neutrophilic granulocyte (10^9^/L)	10.61	1.8–6.3
Lymphocyte (10^9^/L)	1.17	1.1–3.2
Monocyte (10^9^/L)	1.19	0.1–0.6
Eosinophilic granulocyte (10^9^/L)	0.00	0.02–0.52
Basophilic granulocyte (10^9^/L)	0.00	0–0.06
C-reactive protein (mg/L)	82.17	0–6.00
Total protein (g/L)	44	65–85
Albumin (g/L)	27	40–55
Globulin (g/L)	17	20–40
Urea (mmol/L)	11.54	3.1–8
Creatinine (μmol/L)	97.5	57–97
Uric acid (μmol/L)	409	208–428
Potassium (mmol/L)	4.36	3.5–5.3
Sodium (mmol/L)	136	137–147
Chlorine (mmol/L)	104.3	99–110
IL-2 (pg/mL)	1.27	≤7.5
IL-6 (pg/mL)	53.92	≤5.4
IL-10 (pg/mL)	2.27	≤12.9
IFN-γ (pg/mL)	0.66	≤23.1
IL-17 (pg/mL)	3.87	≤21.4
IL-1β (pg/mL)	7.36	≤12.4
IL-4 (pg/mL)	1.22	≤8.56

IL, interleukin; IFN, interferon.

The patient was diagnosed with a right lower limb traumatic fracture accompanied by arterial, venous, and nerve damage. Upon admission, the patient immediately underwent emergency surgery. The patient was identified to be at a high risk of PU development based on the Braden pressure injury scale, and he developed a PU despite preventive measures. On 11 August, on hospital day 14, he developed a PU (stage I) on the right heel. Once the PU developed, a series of intervention methods were added to the prevention protocols: rinsing the wound with physiologic saline, application of collagenase, silver ion dressing, hydrocolloid dressing, and sterile hydrogel dressing, whenever necessary, in order to remove the necrotic tissue. Low-air-loss mattresses, raising the lower limbs, and nutritional supplementation were used to optimize wound healing. Despite these preventive measures, the size and depth of the PU increased with evidence of deep tissue injury in the wound tract. On 9 November, on hospital day 130, the PU progressed to stage IV (2 × 3 cm). Thus, apart from topical wound care, nutritional support, and continuation of preventive care during hospitalization, we undertook the combined use of WJ-MSCs and PRP with intradermal injection in ulcer margins.

### Cell culture and characterization

The clinical grade WJ-MSCs were generated under good manufacturing practice (GMP) conditions with standard operating procedures, as previously described by Zhang et al. (2020). The WJ-MSCs were supplied by the Central Laboratory of Liaocheng People’s Hospital, which has been certified by the National Institutes for Food and Drug Control of China (authorization numbers: SH201900594 and SH201900597). WJ-MSCs were obtained from Wharton’s jelly of the umbilical cords of infants delivered full-term by normal labor. In brief, under sterile conditions, the umbilical cord membrane was stripped, and the umbilical cord’s two arteries and one vein were removed to retain the Wharton’s jelly. The Wharton’s jelly was cut into 1-mm^3^ pieces and then cultured in Dulbecco’s modified Eagle media: Nutrient Mixture F-12 (Gibco, United States) supplemented with 10% fetal bovine serum (FBS) (Gibco, United States). Collected tissues were cultured at 37°C in a 5% CO_2_ incubator with saturated humidity. The culture medium was changed every 3 days. WJ-MSCs were digested and passaged with 0.25% trypsin (Sigma, United States) when they reached 80%–90% confluence.

WJ-MSCs isolated from the Wharton’s jelly of the umbilical cord were examined to confirm their MSC characteristics. Flow cytometry for MSC surface markers (CD90^+^, CD73^+^, CD105^+^, CD45^–^, and CD34^–^) was performed, as previously described by Zhang et al. (2020). Cell surface marker analysis by flow cytometry was performed by incubating WJ-MSCs (2 × 10^5^) with a combination of antibodies: CD90-fluorescein isothiocyanate (FITC, 555595), CD73-phycoerythrin (PE, 550257), CD105-phycoerythrin (PE, 560839), CD45-(APC, 555485), and CD34-phycoerythrin-Cy (PE-Cy, 5555823). These cells were analyzed by fluorescence-activated cell sorting using a Becton-Dickinson instrument (BD, San Diego, CA). The rates of apoptosis and phenotype were analyzed using a FACSCanto Cytometer (BD Biosciences, San Jose, CA) and DIVA software (BD Biosciences, San Jose, CA).

A series of verification tests were conducted before cell therapy, including cell morphology (fibroblast-like adherent cells), cell viability assay (>90% live cells measured by trypan blue assay), purity by FACS (≥95% CD90^+^, CD73^+^, CD105^+^, CD34^−^, and CD45^−^ cells), and endotoxin and *mycoplasma* and sterility testing (−).

#### Blood collection and platelet-rich plasma preparation

Approximately 40 mL of whole venous blood was collected from the forearm vein using 5-mL tubes containing 3.2% sodium citrate, and a 100-μL sample was separated for whole blood platelet count, and the remaining blood was used to produce PRP by a modified two-step centrifugation method, as previously described by Amable et al. (2013). The PRP preparation procedure consisted of two centrifugation steps. First, whole blood was centrifuged at 300 × g for 5 min at 18°C. Under sterile conditions, the upper fraction of each tube was separated, transferred into a new sterile tube, and centrifuged the second time at 700 × g for 17 min at 18°C. After the two centrifugation steps, the upper yellow solution (platelet-poor plasma) was removed, resulting in approximately 5 mL of PRP, which was used to re-suspend WJ-MSCs for subsequent use.

### Ulcer assessment

According to treatment and maintenance procedures for pressure ulcers, the depth, surface area, and tissue type of the ulcer were used as criteria for wound healing. After the pressure wounds were evaluated by the investigators, the wounds with necrotic tissue were removed by an international wound therapist before cell treatment. The debrided ulcers were re-observed and visualized using a mobile phone with a digital camera before the maintenance. The digital picture was taken with standardized settings at a constant distance before the patient was treated. The length, width, and depth of the wound in centimeters were measured and recorded with a disposable wound ruler before maintenance.

### Wound treatment

The WJ-MSCs were cultured and passaged until they were available in the amounts required to be administered to the patient. For cell treatment, cells were harvested, washed three times with PBS, and re-suspended in prepared autologous PRP to be administered within 24 h harvesting of the cells.

Any associated comorbidities of the patient were treated as indicated to optimize and improve the patient’s health and general condition. During treatment, routine laboratory tests were conducted to ensure the stability of the patient’s nutritional status. Before cell therapy, normal saline was used to perform radical debridement of the PU, combined with sharp debridement, if necessary, to remove necrotic tissue and residual foreign bodies from the wound as much as possible. The wound cultures were harvested to exclude local infections. Clinicians performed cell therapy, in which WJ-MSCs (5 × 10^6^) re-suspended in PRP were injected into ulcer margins. Before injection, the wound was locally anesthetized, and the injection point was determined according to the extent of wound involvement. After single-dose administration of WJ-MSCs combined with PRP, the wound was covered with a transparent and nonadherent dressing. During the period of cell treatment, local creams or dressings with potential healing action were not allowed to be used.

### Wound analysis

During the 7-week study period, the treatment’s effectiveness was observed and assessed weekly with wound area measurements from the digital images using the same digital camera on a mobile phone. On days 8, 13, 19, 26, 33, and 40 after cell treatment, wound measurements, including length, width, maximal diameter, and circumference, were taken, and wound closure was examined in a timely manner. Wound closure was considered complete when the entire surface area was covered with epithelial tissue. To underline the reliability of the analysis, this process was performed in a blinded manner by two independent researchers showing minimal interindividual variation, and the average of their measurements was used.

## Results

### Adverse events

No serious adverse events or complications derived from the procedures or treatments were noted within 2 hours after transplantation. In addition, no delayed hypersensitivity or secondary infections were detected after treatment.

### Clinical effects

**FIGURE 1:**
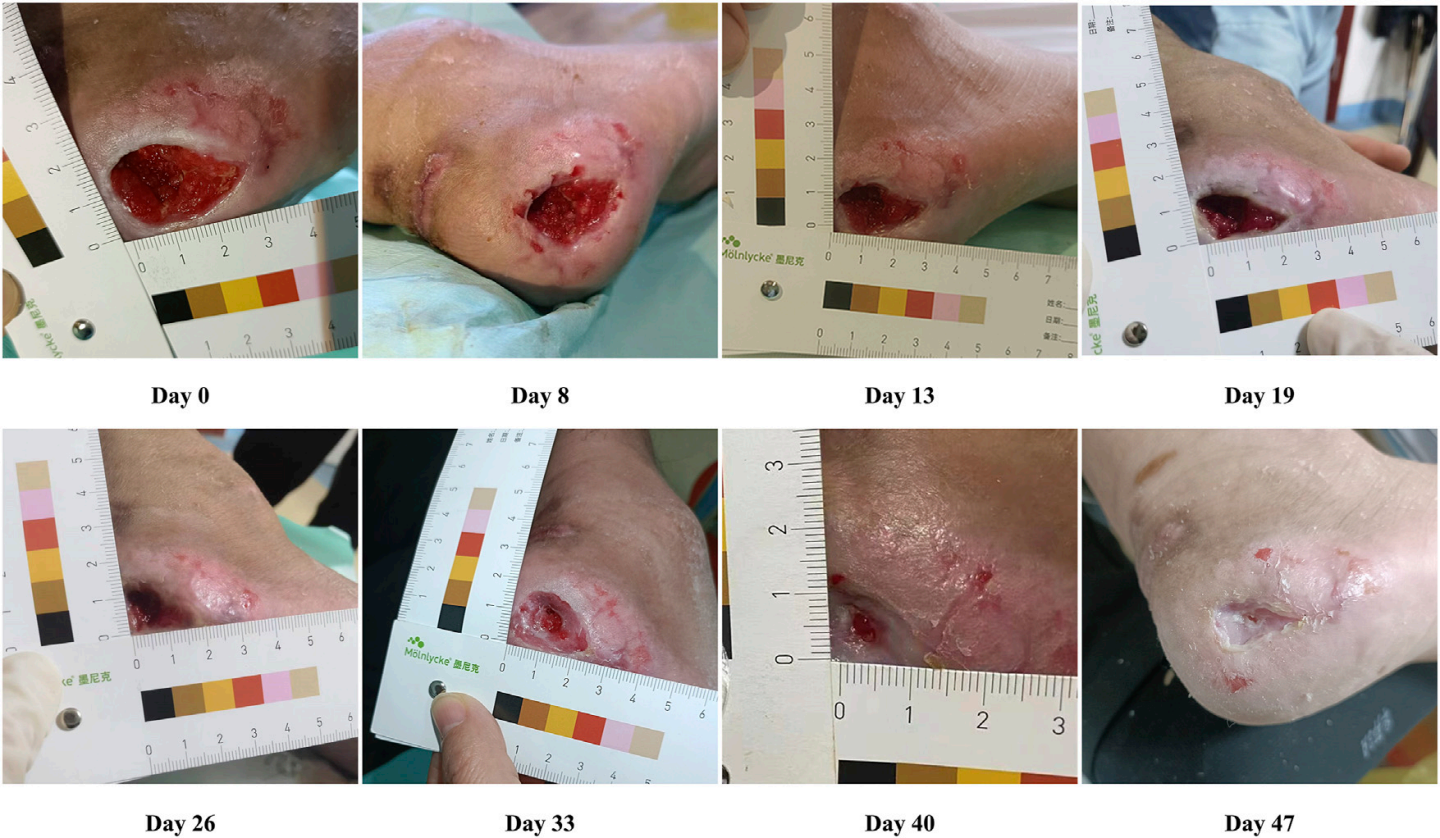
Representative photographs showing the healing progress of pressure ulcers on days 8, 13, 19, 26, 33, and 40 after cell treatment.

Healing progress after the use of WJ-MSCs and PRP in the patient was monitored and evaluated until the wound healed completely. The wound area was observed and assessed before starting therapy. Following that, the treatment with WJ-MSCs and PRP was initiated. A file was created for the patient, which included the healing progress and the date of transition to the granulation and epithelialization stages. The digital images displaying the healing process are shown in Figure 1. The initial wound surface area was 6 cm^2^, and the observation time after cell treatment lasted 47 days. Four days after the WJ-MSCs application, the patient showed progressive healing of the PU. From days 4 to 33, granulation tissue formation and re-epithelialization were clearly observed. On day 33, the wound surface area was 3.68 cm^2^. The ulcer was almost healed completely on day 47, and the pain in the patient’s wound area also decreased. The epithelialization period was found to be quicker than ulcer conventional treatment like negative pressure or advanced moist wound therapies (Roubelakis et al., 2014). Re-epithelialization is a term used to describe the resurfacing of a wound with new epithelium. It is one of the key steps in the process of skin wound repair, which starts 24 h after injury and continues until the healing process covers the entire surface of the wound (Chang et al., 2022). Furthermore, the epithelialization time of skin wounds largely depends on the specific condition of the wound, such as the location, size, depth, microbial contamination, patient health status, genetics, and epigenetics (Sorg et al., 2017). A comparative study of collagenase and hydrogel in maintaining debridement and wound closure in institutionalized adults with pressure ulcers showed all patients (*n* = 3) in the hydrogel group achieved complete epithelialization with a mean of 32.6 days, and 9 out of 10 patients in the collagenase group achieved complete epithelialization with a mean of 45 days (Milne et al., 2012).

During 12 months of follow-up, the resolved ulcers have not recurred again, and no adverse event was reported. To summarize, an accelerated healing process with increased neovascularization in the ulcerative area was identified after cell therapy.

## Discussion

Chronic wounds are one of the major health issues, predominantly affecting older individuals around the world. Chronic dermal ulcers, such as lower limb vein ulcers, diabetes foot ulcers, and PUs, can lead to loss of function and productivity, anxiety, and a decrease in the quality of life (Snyder et al., 2016). Debridement is a key component of wound care, which involves removing necrotic tissue, foreign objects, bacterial growth, and callus from chronic wounds to stimulate the granulation, epithelialization, and proliferation of the wound. The methods of debridement include surgery debridement, autolysis, enzymatic degradation, chemicals, biological surgery, and mechanical dehydration (Waycaster et al., 2018). These methods can be used alone or in combination to optimize the process of debridement. The choice of debridement procedures depends on multiple factors, including patient status, wound characteristics, the likelihood of infection, and available medical expertise, as well as resources (Waycaster and Milne, 2013).

PUs that occur in the hospital are commonly referred to as HAPUs, which are considered an important indicator of the quality of hospital patient care (Spector et al., 2016). Patients who develop HAPUs have longer stays in the hospital than necessary, and they are at a greater risk of infections and are more likely to die (Lyder et al., 2012; Braga et al., 2013). Surgical patients are prone to developing PUs because they often remain immobile for extended periods of time, and their surgical procedures may limit the flow of blood oxygen and nutrition and lead to a decrease in muscle tone. Li et al. (2020) reported the pooled hospital-acquired pressure injuries rate among 1,893,593 patients was 8.4%, and the most common stages were stage I (43.5%) and stage II (28.0%). Most body sites affected by ulcers in surgical patients are commonly found on the sacrum, coccyx, heel, and back of the head. HAPUs can be partially prevented by postoperative turning and early mobilization after the surgical procedure, minimizing shear force and pressure through appropriate positioning, and improving blood and oxygen flow (Feuchtinger et al., 2005; Spector et al., 2016). Prevention of PUs is one of the most important indicators of the quality of healthcare services. However, it is noteworthy that the frequency of HAPUs for surgical patients is still high.

In this study, we reported a surgical patient who developed an ulcer during hospitalization. This study evaluated the safety and efficacy of intradermal injection of WJ-MSCs re-suspended in PRP in improving and accelerating wound healing. The obvious side effects are not found in the treatment of cell transplantation, indicating it was well-tolerated and safe. Our results showed that the administration of WJ-MSCs resulted in improved granulation, facilitated re-epithelialization, and accelerated wound closure. Compared with traditional treatment, MSC treatment can shorten the healing time of ulcers. The healing time of PU varies and largely depends on the stage of the PU and the patient’s physiologic condition. Moreover, prolonged non-healing of ulcers during hospitalization can increase hospitalization costs. HAPUs can add approximately 44% to the cost of major surgical hospital stays, but the amount varies depending on the total cost during hospitalization (Spector et al., 2016). A study of 19,889 older adults in 51 nursing homes found that 75% of PUs (stage II) and 17% of PUs (stages III or IV) healed in 8 weeks, and it is worth noting that 23% of PUs (stage II) and 48% of PUs (stage IV) had not healed after 1 year (Brandeis et al., 1990). Although surgical treatment for pressure ulcer patients (stages III and IV) typically leads to faster healing, roughly 6 weeks of bed rest is still required, and the recurrence rates of ulcers are high (Feldman and McCauley, 2018).

**FIGURE 2:**
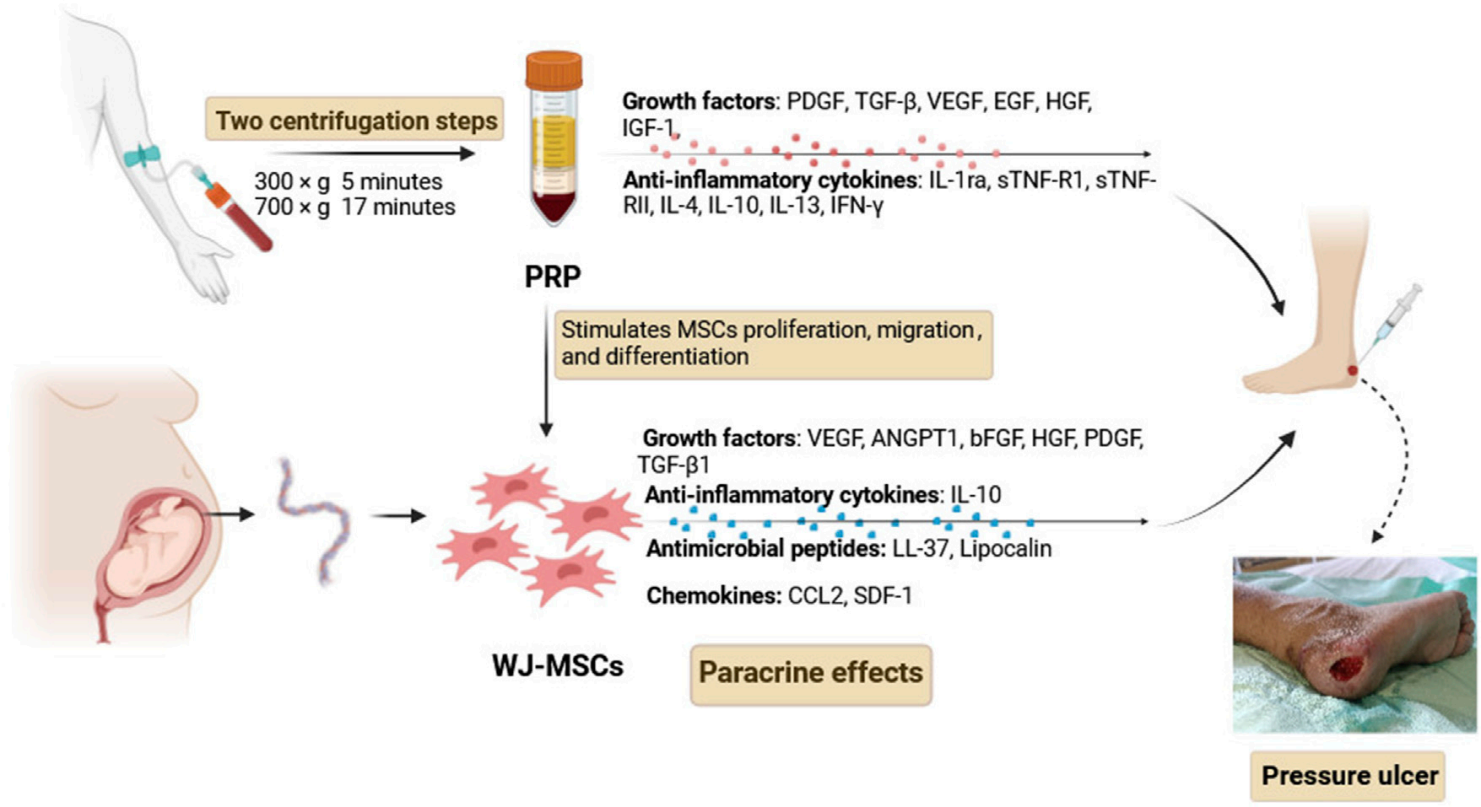
Mechanisms of WJ-MSCs and PRP treatment for wound healing. Abbreviations: PDGF, platelet-derived growth factor; TGF-β, transforming growth factor β; EGF, epidermal growth factor; HGF, hepatocyte growth factor; IGF-1, insulin growth factor-1; VEGF, vascular endothelial growth factor; ANGPT1, angiopoietin 1; bFGF, basic fibroblast growth factor; IL-1ra, interleukin-1 receptor antagonist; sTNF-R1, soluble tumor necrosis factor receptor 1; sTNF-RII, soluble tumor necrosis factor receptor II; IL-4, interleukin-4; IL-10, interleukin-10; IL-13, interleukin-13; IFN-γ, interferon-γ; CCL2, chemokine (C-C motif) ligand 2 (CCL2); SDF-1, stromal cell-derived factor-1.

MSCs represent an attractive stem cell source for tissue regeneration in clinical applications, which have been demonstrated to improve wound healing through re-epithelialization, increased angiogenesis, and granulation tissue formation. At the wound site, MSCs play an active role in modulating the inflammatory environment, enhancing angiogenesis, and promoting the migration of keratinocytes and recruitment of other host cells, which can contribute to the generation of granulation tissue, promote angiogenesis and epithelialization, and attenuate scar formation (Kamolz et al., 2014; Laverdet et al., 2014). The mechanisms of MSC-based treatment for wound healing have not been fully elucidated, but it is widely believed that the treatment effects mainly depend on their paracrine effects (Figure 2). MSCs can secrete a large number of soluble factors, such as growth factors, anti-inflammatory cytokines, antimicrobial peptides, chemokines, and exosomes, to enhance the growth, survival, and function of wound repair cells. To determine the therapeutic potential of MSCs for PUs, several clinical trials have shown improved and accelerated wound healing after the transplantation of MSCs (Yoshikawa et al., 2008; Sarasúa et al., 2011). PRP is widely used in wound healing, and it provides a release of multiple functional growth factors which are beneficial for skin wound healing and cell proliferation, including platelet-derived growth factor (PDGF), transforming growth factor β (TGF-β), vascular endothelial growth factor (VEGF), epidermal growth factor (EGF), hepatocyte growth factor (HGF), insulin growth factor 1 (IGF-1), and interleukins (Marx, 2001; Arnalich et al., 2016). Moreover, PRP can boost the potency of transplanted MSCs by serving as a cellular scaffold used in clinical applications. When administered as a co-adjuvant to MSCs, PRP stimulates cell proliferation, preserves differentiation potential, and does not affect any lineage differentiation in a controlled, nontumorigenic manner (Mahmoudian-Sani et al., 2018). PRP can serve as a regulatory factor for cell migration and wound healing, promoting the migration of MSCs and the wound-healing process. The possible mechanism involved in the increased growth of the MSCs is that PRP leads to an increase in the number of cells at the transplantation site by increasing cell proliferation and utilizing the MSCs further. The combined effect of MSCs and PRP has been evaluated in a preclinical trial by Lian et al. (2014), which showed that wound healing rates were significantly higher in the BM-MSCs plus PRP group than in the other groups. Their results found that the combined administration of BM-MSCs and PRP can accelerate wound healing by enhancing cell proliferation, increasing angiogenesis, affecting the infiltration response, and inducing TGF-β1 expression.

The present study is still subject to several limitations. First, the number of patients recruited in this study is small, and only one patient was enrolled. A placebo group was not designed due to ethical reasons. Therefore, in future clinical trials, using standard wound care as the control group to validate the safety and efficiency of MSC-based therapy is reasonable. Second, ulcer measurements with a ruler and multiplying to calculate the total surface area of the ulcer are more likely to lead to an overestimation of the calculated surface. Efforts to minimize potential biases in wound measurements were made by two independent researchers. In addition, standardizing photographs is very difficult because the distance between the photographs recorded and the injury varied. Despite an extensive search across databases, there are many differences in clinical trials that lead to direct comparisons of the outcomes between different studies, including cell sources, doses, administration route, and wound types and staging. Due to the limited number of randomized controlled clinical trials conducted so far, there is an urgent need for high-quality clinical research in the future, especially multicenter, randomized, double-blind, and placebo-controlled clinical trials.

## Conclusion

The treatment of WJ-MSCs together with autologous PRP improves and accelerates PU healing in comparison with traditional treatment. WJ-MSCs, together with PRP, may offer a promising treatment option for wound healing. A deeper understanding of the interaction mechanism between WJ-MSCs and PRP, including the migration and homing of WJ-MSCs to the tissue of injury, and understanding how autologous PRP regulates and supports the activation and differentiation of WJ-MSCs during the wound-healing process will help optimize treatment plans for patients with chronic wounds. In the future, further studies, especially large-scale and multicenter studies, are necessary to confirm the efficacy of using WJ-MSCs or PRP alone or a combination of both in the treatment of PUs and determine the optimal application plan for patients to improve survival and quality of life.

## Data Availability

The original contributions presented in the study are included in the article/Supplementary Material; further inquiries can be directed to the corresponding authors.
